# Type I interferon receptor-independent and -dependent host transcriptional responses to mouse hepatitis coronavirus infection *in vivo*

**DOI:** 10.1186/1471-2164-10-350

**Published:** 2009-08-03

**Authors:** Matthijs Raaben, Marian JA Groot Koerkamp, Peter JM Rottier, Cornelis AM de Haan

**Affiliations:** 1 Virology Division, Department of Infectious Diseases and Immunology, Faculty of Veterinary Medicine, Utrecht University, Yalelaan 1, 3584 CL Utrecht, the Netherlands; 2 University Medical Center Utrecht, PO Box 85060, 3508 AB Utrecht, The Netherlands

## Abstract

**Background:**

The role of type I IFNs in protecting against coronavirus (CoV) infections is not fully understood. While CoVs are poor inducers of type I IFNs in tissue culture, several studies have demonstrated the importance of the type I IFN response in controlling MHV infection in animals. The protective effectors against MHV infection are, however, still unknown.

**Results:**

In order to get more insight into the antiviral gene expression induced in the brains of MHV-infected mice, we performed whole-genome expression profiling. Three different mouse strains, differing in their susceptibility to infection with MHV, were used. In BALB/c mice, which display high viral loads but are able to control the infection, 57 and 121 genes were significantly differentially expressed (≥ 1.5 fold change) upon infection at 2 and 5 days post infection, respectively. Functional association network analyses demonstrated a strong type I IFN response, with Irf1 and Irf7 as the central players. At 5 days post infection, a type II IFN response also becomes apparent. Both the type I and II IFN response, which were more pronounced in mice with a higher viral load, were not observed in 129SvEv mice, which are much less susceptible to infection with MHV. 129SvEv mice lacking the type I interferon receptor (IFNAR-/-), however, were not able to control the infection. Gene expression profiling of these mice identified type I IFN-independent responses to infection, with IFN-γ as the central player. As the BALB/c and the IFNAR-/- 129SvEv mice demonstrated very similar viral loads in their brains, we also compared their gene expression profiles upon infection with MHV in order to identify type I IFN-dependent transcriptional responses. Many known IFN-inducible genes were detected, several of which have previously been shown to play an important protective role against virus infections. We speculate that the additional type I IFN-dependent genes that we discovered may also be important for protection against MHV infection.

**Conclusion:**

Transcriptional profiling of mice infected with MHV demonstrated the induction of a robust IFN response, which correlated with the viral load. Profiling of IFNAR-/- mice allowed us to identify type I IFN-independent and -dependent responses. Overall, this study broadens our present knowledge of the type I and II IFN-mediated effector responses during CoV infection *in vivo*.

## Background

Cytokines are key regulators that dictate many aspects of innate and adaptive immunity. Induction of type I interferons (IFNs), a well-known subset of cytokines with antiviral activity, is triggered by a selection of cellular pattern recognition receptors, including TLRs (Toll-like receptors), RIG-I (retinoic acid-inducible gene I), and MDA5 (melanoma differentiation-associated protein 5). These receptors are activated in response to a range of pathogen-specific factors, which includes double-stranded RNA produced during virus infection [1,2]. Secreted type I IFNs (i.e. IFN-α and IFN-β), subsequently induce an antiviral transcription program in the infected cell as well as in adjacent cells, thereby magnifying the "danger" signal and protecting against the infection.

The role of type I IFNs in controlling coronavirus (CoV) infections is not well understood. A number of studies has shown that CoVs, like the mouse hepatitis virus (MHV) and the severe acute respiratory syndrome (SARS)-CoV, are poor inducers of type I IFNs in cell culture, and even escape from detection by cytoplasmic pattern recognition receptors [3-8]. Consistently, virus-encoded IFN antagonistic functions have been described for both MHV and SARS-CoV [9,10]. *In vivo*, however, MHV infection appeared to induce the production of IFN-α in plasmacytoid dendritic cells (pDCs) by a TLR7-dependent mechanism [11]. Moreover, MHV infections of primary neuronal cultures and of the central nervous system (CNS) induced IFN-β gene expression, indicating that the production of type I IFNs *in vivo *is not limited to pDCs [12,13]. Furthermore, neuronal cultures infected with MHV exhibited increased expression of several type I IFN-induced transcription factors [14]. More recently, Roth-Cross and co-workers reported that macrophages and macrophage-like microglia cells produce IFN-β in the CNS of MHV-infected mice in a MDA5-dependent manner [15].

Several studies have demonstrated the importance of the type I IFN response in controlling MHV infection *in vivo*. The exogenous delivery of type I IFNs was shown to inhibit MHV infection of and spread to the mouse brain [16,17]. Consistently, infection of mice lacking the functional type I IFN receptor (IFNAR-/-) with MHV resulted in increased viral replication and extended tissue tropism [11,17,18]. Although many type I IFN-responsive genes have been identified [19], the protective effectors against MHV infection are yet unknown [20].

In order to get more insight into the antiviral gene expression induced in the brains of MHV-infected mice, we performed whole-genome expression profiling. Three different mouse strains (BALB/c, 129SvEv and IFNAR-/- 129SvEv mice), differing in their susceptibility to infection with MHV, were used. Previously, we have observed that 129SvEv mice are significantly more resistant to infection via the intranasal route than BALB/c mice [17]. The reason for the significant difference in susceptibility is not known, but may be related to different antiviral immune responses in these two mouse strains. Furthermore, gene expression profiling of 129SvEv mice lacking the type I IFN receptor, which are not able to control the MHV infection [11], allowed us to identify type I IFN-independent transcriptional responses.

## Results & discussion

**Figure 1:**
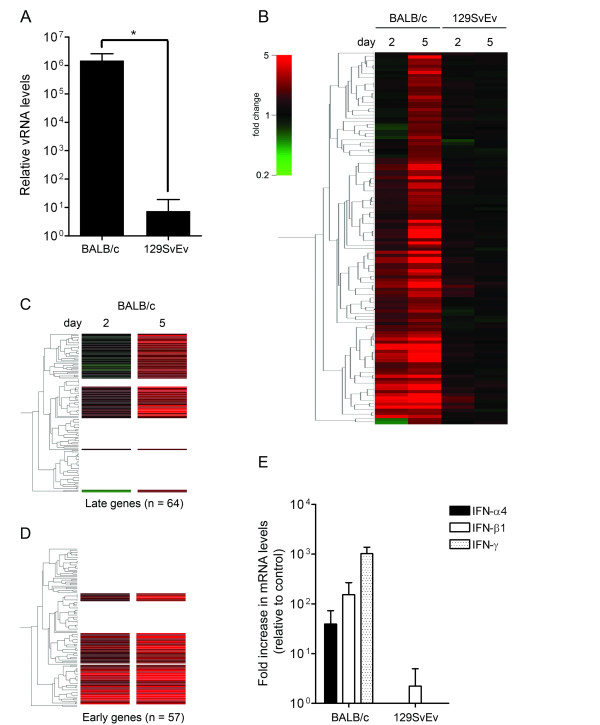
**Genome-wide expression profiling of the brains of BALB/c and 129SvEv mice infected with MHV**. BALB/c and 129SvEv mice were intranasally inoculated with PBS (control) or with MHV-A59. At day 2 and 5, mice (n = 4) were sacrificed and the brains and livers were harvested. The PBS-groups (n = 4) were also sacrificed at 5 days post inoculation. (A) Viral RNA (vRNA) levels within the brain were determined at 5 days post inoculation by quantitative RT-PCR targeting MHV-specific sequences. Standard deviations are indicated (*P < 0.0001). (B) Microarray analysis was performed as described in the *Material & Methods *section. The PBS-inoculated BALB/c or 129SvEv mice were taken as reference. Based on the significant alterations in gene expression (≥ 1.5 fold change cut-off) within the brains of BALB/c mice at 5 days post infection, a cluster analysis (standard correlation) was performed resulting in the indicated gene tree (n = 121). The different conditions (i.e. mouse strains and day post infection) are indicated. (C-D) From the the gene tree shown in panel B, clusters representing "early" and "late" genes could be identified. For detailed information see the text and Additional file 1. (E) The IFN-α4, IFN-β1, and IFN-γ mRNA levels were determined by quantitative RT-PCR. The fold changes after infection with MHV relative to the PBS-inoculated animals are shown. Standard deviations are indicated.

We started by comparing the whole-genome expression profiles in the brains of the BALB/c and the 129SvEv mice upon infection with MHV. To this end, mice were inoculated intranasally with 10^6 ^TCID_50 _of MHV strain A59 or with PBS (control). Groups of mice (n = 4) were sacrificed at 2 and 5 days post inoculation after which the brains were harvested and total RNA was isolated. The extent of virus replication was determined by quantitative reverse transcriptase (RT)-PCR targeting MHV-specific RNA sequences as described earlier [21]. Previously, we demonstrated that the viral RNA load correlates well with viral infectivity in tissue homogenates [17]. While no viral RNA could be detected yet at 2 days post inoculation (data not shown), viral RNA was observed in the brain of both mouse strains at day 5 (Figure 1A). As expected, the BALB/c mice displayed a much higher viral RNA load than the 129SvEv mice.

Next, the RNA extracts were processed for microarray analysis using the PBS-inoculated groups as the reference. In total, 57 and 121 genes were significantly differentially expressed (≥ 1.5 fold change) in BALB/c mice at 2 and 5 days post infection, respectively. In contrast, in the 129SvEv mice, no significant induction of gene expression was observed. The results are depicted in Figure 1B as a gene tree that was built based on the genes with a significantly altered expression level in BALB/c mice at 5 days post infection (i.e. expression-based cluster analysis). From these data we were able to identify host genes, the increased expression (≥ 1.5 fold) of which could already be detected at day 2 (i.e. early genes; Figure 1C) or only at day 5 (i.e. late genes; Figure 1D). The group of early-induced transcripts contained many IFN-inducible genes, including the well-known interferon regulatory factor 7 (*Irf7*), signal transducer and activator of transcription 1 (*Stat1*), and 2'-5' oligoadenylate synthetase (*Oas*) genes (Additional file 1A). Within the cluster of "late" genes (Additional file 1B) several chemokines (i.e. *Ccl2*, *Ccl5*, *Ccl7*, *Cxcl9*, and *Cxcl10*) could be identified.

**Figure 2:**
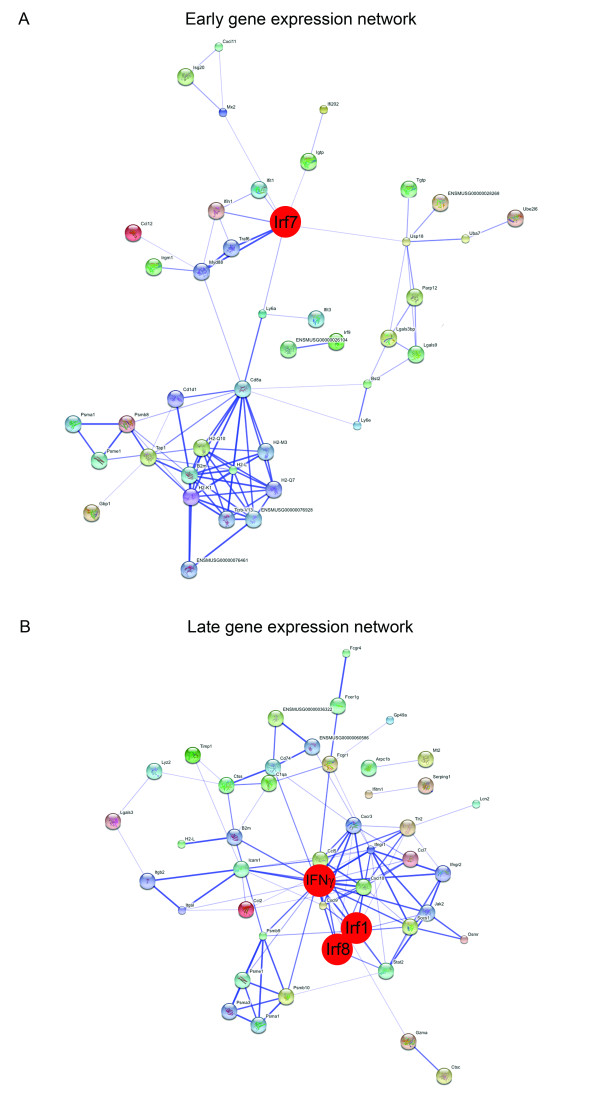
**Early and late transcriptional responses to infection with MHV**. (A) The early gene expression network. The "early" genes listed in Additional file 1A (n = 57) were subjected to functional association network analysis by using the public STRING 8.0 database http://string.embl.de/. Indicated is the confidence view of the analysis. Stronger associations are symbolized by thicker lines. (B) The late gene expression network. The "late" genes listed in Additional file 1B (n = 64) were subjected to functional association network analysis as described above. In both panels, the key players in the network (i.e. Irf7 for panel A and IFN-γ, Irf1, and Irf8 for panel B) are indicated in red.

Next, in order to construct a functional association network, we applied the STRING 8.0 software [22] to the list of proteins encoded by the "early" and "late" genes. We also included known interactors of our hits in this analysis, while proteins that did not demonstrate any known interactions were excluded for clarity. The results are shown in Figure 2A and 2B. Functional association network analysis of the proteins encoded by the "early" genes revealed two main modules. One module contained several proteins involved in antigen presentation, while the other module contained numerous proteins involved in the type I IFN response. The key player in this latter module appeared to be Irf7, which is the master regulator of type I IFN-dependent responses [23]. Functional association network analysis of the proteins encoded by the "late" genes revealed a large network of proteins involved in host-pathogen interactions. Although the microarray analyses did not reveal the induction of IFN-γ gene expression itself, IFN-γ appeared at a central position in the network. In addition, the induction of a type I IFN response was also evident from this network as demonstrated by the presence of the transcription factors Irf1 and Irf8, both of which demonstrated elevated mRNA levels upon MHV infection. In conclusion, these results demonstrate that MHV infection induces a robust IFN response both at 2 and 5 days post infection, in which the transcription factors Irf7, Irf1, and Irf8 appear to be the key players. At 5 days post infection, a type II IFN response also becomes apparent.

To confirm and extend these observations, we next analyzed the induction of type I and II IFN gene expression (i.e. IFN-α4 and IFN-β1, and IFN-γ, respectively) by using quantitative RT-PCR. In agreement with the microarray expression profiles, significant induction of these type I and II IFNs could only be detected in the MHV-infected BALB/c animals (Figure 1E). The observation that the BALB/c mice, unlike the 129SvEv mice, exhibited abundant expression of IFN-responsive genes upon MHV infection appears counter intuitive as the 129SvEv mice are much more resistant to the infection than the BALB/c mice. Apparently, the resistance of 129SvEv mice to MHV infection is not controlled by a more robust IFN response. The reason for the observed difference in susceptibility between the different mouse strains after intranasal inoculation is not known. MHV-A59 was recently shown to replicate efficiently in the liver of 129SvEv mice after intraperitoneal inoculation [11]. Interestingly, the resistance of 129SvEv mice after intranasal inoculation is not restricted to infection with MHV, as it was also observed for vesicular stomatitis virus [24].

**Figure 3:**
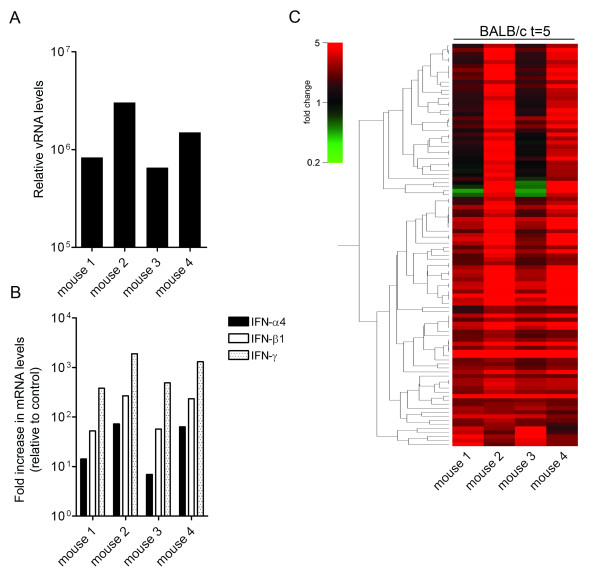
**Induction of gene expression correlates with the viral load**. vRNA (panel A) and IFN-α4, IFN-β1, and IFN-γ mRNA (panel B) levels within the brains of the individual BALB/c mice (mouse 1–4) at 5 days post infection were determined as described in the legend of Figure 1. (C) Microarray data analysis of the individual BALB/c mice (mouse 1–4). The gene tree shown (n = 96) is based on the significant alterations at 5 days post infection while applying an expression cut-off (≥ 2.0 fold). For detailed information see Additional file 2.

The microarray expression profiles described above suggested that the induction of an IFN response correlates with the viral load within the brain. To confirm this, we examined the data of the individual BALB/c mice at 5 days post infection in more detail. Clearly, the animals with the highest viral loads (mouse 2 and 4; Figure 3A), also displayed significantly higher levels of induction of type I and II IFN expression (Figure 3B). Likewise, the amplitude of the gene expression profiles (Figure 3C and Additional file 2) of the individual mice also correlated with the viral loads in the brain. These observations are in agreement with results obtained by the profiling of SARS-CoV-infected macaques [25]. Also in that study a positive correlation between virus load and the induction of gene expression was observed. A few genes (n = 6), including *ISG20*, showed an inverse correlation with the viral load. We currently have no explanation for this observation as expression of *ISG20 *is known to be induced by type I IFNs [26,27]. Interestingly, ISG20 has been shown to exhibit antiviral activity against other viruses [28,29].

**Figure 4:**
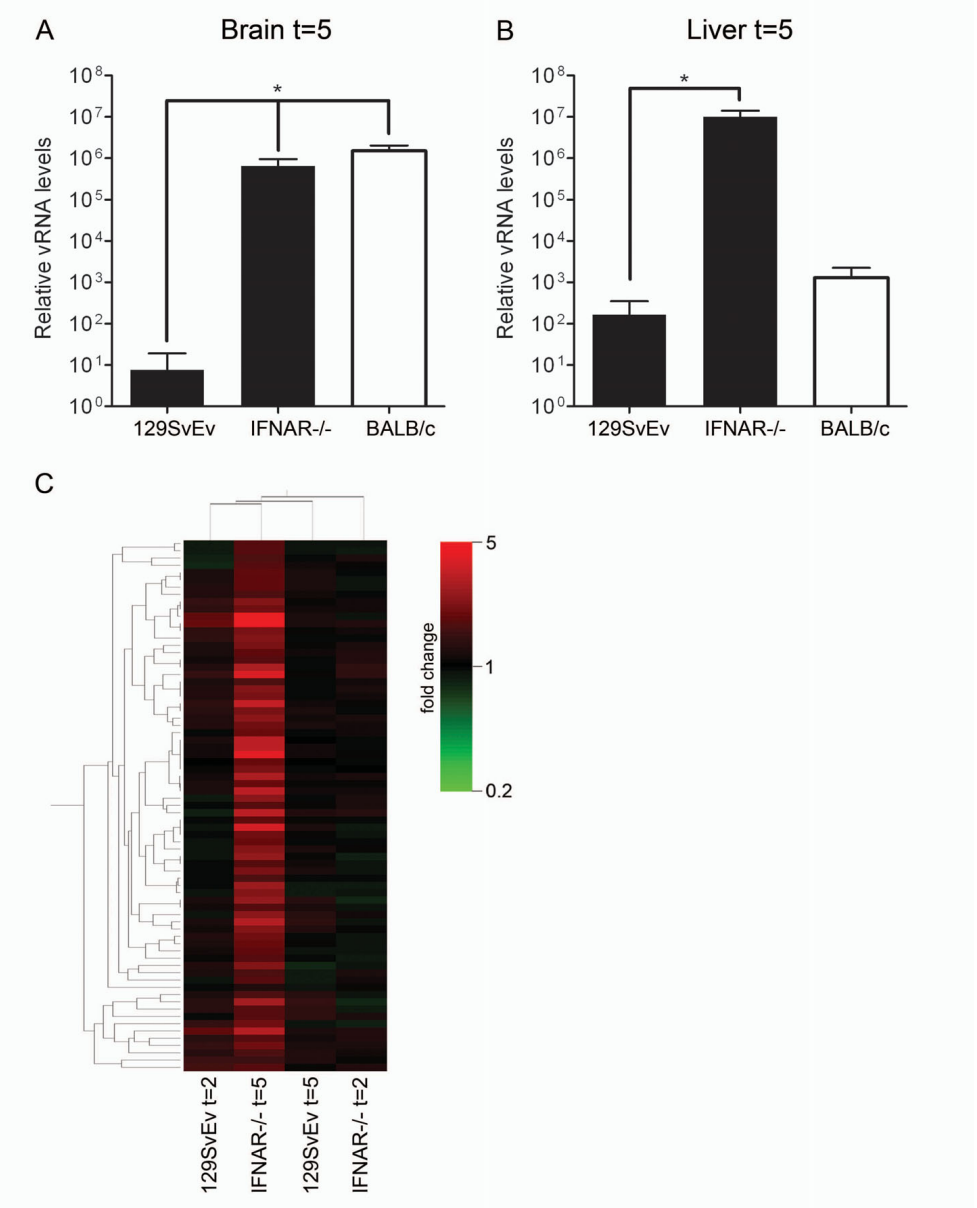
**The type I IFN receptor-independent expression profile within the brains of IFNAR-/- mice after MHV infection**. IFNAR-/- 129SvEv mice were intranasally inoculated with 10^6 ^TCID_50 _of MHV-A59 or treated with PBS (control). At day 5, mice (n = 4) were sacrificed and the brains and livers were harvested. (A and B) The vRNA levels within brains and livers were determined as described in the legend of Figure 1. Standard deviations are indicated (*P < 0.0001). Also depicted are the vRNA levels for the parental 129SvEv mice and BALB/c mice. (C) Total RNA samples obtained from the brains of PBS- or MHV-inoculated IFNAR-/- mice were processed for microarray analysis as described in the legend to Figure 1. Based on the significant alterations (≥ 1.5 fold change cut-off) in gene expression within the brains of the IFNAR-/- mice at 5 days post infection (n = 73) a gene tree was build. The different conditions (i.e. mouse strain and day post infection) are indicated. See Additional file 3 for details. Note that the different conditions are also clustered according to their similarities in the complete gene expression profile.

To study the role of type I IFN-independent and -dependent gene expression in the control of MHV infection *in vivo *in more detail, we next made use of the IFNAR-/- mice [30]. These mice are highly susceptible to MHV infection as compared to the parental 129SvEv mice [11,17]. Indeed, when these mice were inoculated intranasally with 10^6 ^TCID_50 _of MHV-A59, viral RNA levels in their brains became much higher than in animals from the parental strain at 5 days post infection (Figure 4A). Interestingly, at this time point the viral RNA levels in the IFNAR-/- mice were comparable to those in the brains of the BALB/c mice. However, efficient dissemination of the infection, resulting in high viral loads in the liver as determined by quantitative RT-PCR, was only observed in the IFNAR-/- mice and not in the wild-type mice, which displayed viral RNA levels just above background (Figure 4B). Thus, in agreement with previous studies, a type I IFN-dependent response is required to inhibit virus dissemination [11,15].

Whole-genome expression profiling of brains of the IFNAR-/- mice revealed the significantly induced expression of 73 genes (≥ 1.5 fold) at 5 days post infection. In contrast, at day 2, hardly any alterations in gene expression could be detected in these knock-out mice (Additional file 3). Figure 4C shows an expression-based cluster analysis of these 73 genes for the wild-type and IFNAR-/- mice. Comparison of the complete expression profiles of these mice revealed that the transcriptional profile at day 5 in the IFNAR-/- mice has a larger similarity with the profile at day 2 of the parental 129SvEv mice than with that of the knock-out mice at day 2 post infection (Figure 4C). This observation may suggest the presence of an early host response to infection with MHV in the parental mice, even though no significant induction (≥ 1.5 fold) of gene expression could be detected (Figure 1B). Such a response, may not be evident in transcriptional profiles of whole organs, but might only be apparent at the cellular level. We speculate that early decisive events are happening in initial target cell populations such as DCs and macrophages [31]. These responses could prevent extensive viral replication very early after infection, thereby reducing subsequent type I IFN responses.

As the knock-out mice lack a functional type I IFN receptor, the upregulation of gene expression observed in these mice apparently occurs independently of type I IFN signalling. Not much is known yet about type I IFN-independent responses to infection. The observation that the transcriptional upregulation of *Irf1 *was independent of type I IFN signalling is consistent with the notion that IFN-γ can also induce expression of this gene [32,33]. Likewise, we also observed increased transcription of *Ifitm1 *and *Ifitm3 *independent of type I IFN signalling, again corresponding with the literature [34,35]. Interestingly, the expression of various genes encoding proteins involved in antigen presentation (i.e. *H2*, *B2m*, *Psmb8*, *Psmb9*, and *Ctss*) was also increased in the absence of type I IFN signalling. *Psmb8 *and *Psmb9 *encode immunoproteasome subunits which facilitate antigen presentation to CD8^+ ^T cells after virus infection, a process that is primarily regulated by IFN-γ [36]. Furthermore, also the expression of the major histocompatibility complex class II (MHC II) invariant chain, also called CD74 [37], was increased upon infection of the knock-out mice. These data are in agreement with the observation that the induction of genes involved in antigen processing is independent of STAT1 activation by IFN-α [38]. We also observed the transcriptional upregulation of the 3 isoforms of metallothionein (*Mt1*, *Mt2*, and *Mt3*), which encode proteins known to scavenge toxic metals [39]. The induction of these genes, which was not apparent in either wild-type mice, could reflect an acute-phase reaction in the brain of MHV-infected IFNAR-/- mice, which likely contributes to pathogenesis as has been shown for other viruses [40-42].

**Figure 5:**
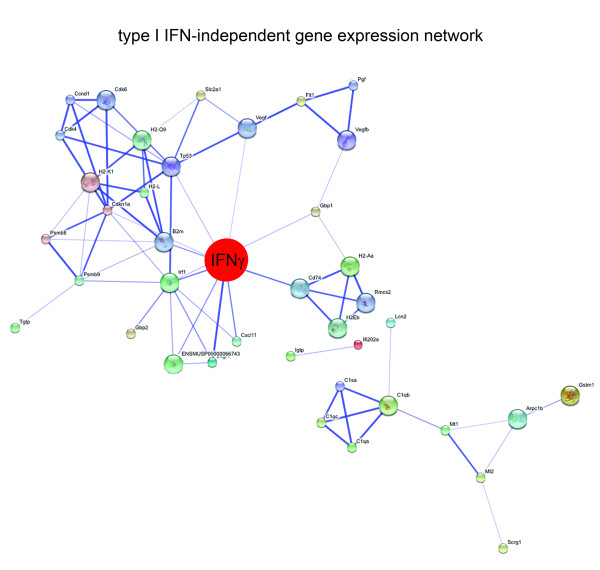
**The type I IFN-independent gene expression network**. The genes listed in Additional file 3 (n = 73) were subjected to functional association network analysis by using the STRING 8.0 database as described in the legend of Figure 2. The key player in the network, IFN-γ, is indicated in red.

We constructed a functional association network by applying the STRING 8.0 software [22] to the list of proteins encoded by the type I IFN-independent genes (Additional file 3). We also included known interactors of our hits in this analysis, while proteins that did not demonstrate any interactions were again excluded for clarity. The result is shown in Figure 5. The analysis revealed IFN-γ as the central player in the type I IFN-independent antiviral network as this protein appeared to link a number of smaller modules. The induction of IFN-γ gene expression could be confirmed using quantitative RT-PCR (data not shown). The finding that IFN-γ-mediated transcriptional responses are not dramatically affected in the absence of type I IFN signalling is in agreement with reports referred to above and with a recent publication by Ireland et al. [18], which shows that IFN-γ expression is significantly induced in the CNS of MHV-infected IFNAR-/- mice. While the production of IFN-γ by NK cells plays a major role in the protection against infection with MHV [43-47], the IFN-γ-mediated transcriptional responses that we observed were not protective against acute MHV infection in the IFNAR-/- mice.

Several studies have shown that MHV [11,15,17] as well as several other viruses [48-50] replicate to much higher levels (up to 10^5 ^fold difference) in IFNAR-/- mice than in their wild-type counterparts. In this study we show that a strong correlation exists between the amplitude of type I and II IFN host responses with the viral load. The huge differences in virus replication between wild-type and IFNAR-/- mice therefore do not permit a fair comparison between gene expression profiles of these mice, with the aim of identifying type I IFN-dependent responses. Indeed, as no significant gene expression is observed in the wild-type 129SvEv mice, a comparison with the expression profile of the IFNAR-/- mice only provides information about type I IFN-independent and not IFN-dependent responses. We now observe, in agreement with our previous study, that the brain of BALB/c and IFNAR-/- 129SvEv mice contain very similar MHV loads at day 2 and 5 post infection [17]. Since the type I IFN-responsive pathway is very well conserved among many different species [51], we considered it acceptable to compare the gene expression profiles of these mice with the aim of identifying type-I IFN-dependent responses, although comparing transcriptional profiles of wild-type and IFNAR-/- mice from a different genetic background should obviously be done very cautiously. Ideally, a comparison between wild-type BALB/c and IFNAR-/- BALB/c mice would have been more accurate. While the induced expression of a number of genes was similar for the two mouse strains (i.e. type I IFN signalling-independent gene-expression), that of other genes was only observed in the BALB/c mice (i.e. tentative type I IFN signalling-dependent gene expression). The expression of yet other genes appeared to be partially dependent of type I IFN signalling: increased expression of these genes was observed in the IFNAR-/- mice, but much more so in the BALB/c mice.

Genes, the expression of which was upregulated (≥ 1.5 fold) in the BALB/c mice but not significantly changed in the IFNAR-/- mice upon infection with MHV, were tentatively designated as type I IFN-dependent. Genes, the transcriptional upregulation of which was at least 2 times higher in the BALB/c mice than in the IFNAR-/- mice, were also added to the list of tentative type I IFN-dependent genes. As expected, this set of genes (n = 82) contained many known IFN-responsive genes like *Isg20*, *Ifit1*, *Ifit3*, *Isgf3g, Mx2 and Ube1l *(Additional file 4). Functional association network analyses showed Irf1 and Irf7 to be the key players in the network (Additional file 5). Several of the tentative type I IFN-dependent genes (including *Mx2 *and *Ube1l*) have previously been shown to play an important protective role against virus infections [52-56]. We speculate that other genes present in this list may also be important for full protection against MHV infection.

## Conclusion

Transcriptional profiling of mice infected with MHV demonstrated the induction of a robust IFN response, which correlated with the viral load. Profiling of IFNAR-/- mice allowed us to identify type I IFN-independent and -dependent responses. Overall, this study broadens our present knowledge of the type I IFN-mediated effector responses during CoV infection *in vivo*.

## Methods

### Mouse infection experiments

6–8 week old BALB/c were obtained from Charles River Laboratories, while type I IFN receptor knock-out mice (IFNAR-/-) [30] and the parental 129SvEv mice were obtained from B&K Universal Ltd. Mice were inoculated intranasally with 10^6 ^TCID_50 _of MHV strain A59 and sacrificed at the indicated time-points for organ dissection. Control animals were treated with PBS. The study protocol was approved by the animal ethics committee of the Utrecht University, and all experiments were performed in accordance with accepted institutional and governmental policies.

### Tissue homogenization and isolation of total RNA

Whole brains and livers were dissected from the MHV-infected and control mice. The tissues were added to Lysing Matrix D tubes (MP Biomedical), containing 1 ml of RNA*pro*™ solution (Q-BIOgene), and processed using a FastPrep instrument (MP Biomedical). The tissues were homogenized at 6,000 rpm for 40 sec and immediately placed on ice. Subsequently, the homogenates were centrifuged at 14,000 rpm for 10 minutes at 4°C and supernatants were harvested and stored at -80°C. Total RNA was isolated from the homogenates using the TRIzol reagent (Invitrogen) according to the manufacturer's protocol. RNA was further purified using the RNeasy mini-kit with subsequent DNaseI treatment on the column (Qiagen). RNA integrity was determined by spectrometry and by a microfluidics-based platform using a UV-mini1240 device (Shimadzu) and a 2100 Bioanalyzer (Agilent Technologies), respectively.

### Quantitative RT-PCR

1 μg of total RNA was reverse transcribed into cDNA using 0.5 μM oligo(dT) primers and 20 U of M-MuLV-Reverse transcriptase (Fermentas) in a total reaction volume of 20 μl for 1 h at 37°C. Subsequently, gene expression levels of type I and II IFNs (i.e. IFN-α4 [NM_010504.2], IFN-β1 [NM_010510.1], and IFN-γ [NM_008337.3], respectively), were measured by quantitative PCR using Assay-On-Demand reagents and equipment (PE Applied Biosystems), according to the manufacturer's instructions. The quantitative PCR reactions were performed in a total reaction volume of 20 μl containing 10 μl Taqman^® ^Universal PCR Master Mix (2×), 5 μl cDNA, 1 μl TaqMan^® ^Gene Expression Assay Mix (20×), and 4 μl water using an ABI Prism 7000 sequence detection system under the following conditions: 95°C for 10 mins, followed by 40 cycles of 95°C for 15 secs and 60°C for 1 min. For all assays, we performed "no-RT" (reaction using total RNA as the substrate) and "no template" (reaction using water as the substrate) controls. In both cases, omitting cDNA from the reaction resulted in a lack of PCR product generation. All assays were analyzed with ABI Prism 7000 Software v1.2.3f2 (PE Applied Biosystems). The comparative Ct-method was used to determine the fold change for each gene (primer efficiencies were similar for both the endogenous control primer set and genes of interest primer sets [data not shown]). Note that the Ct values of all samples were within the limits of the standard curves (data not shown). The housekeeping gene GAPDH (NM_008084.2) was used as a reference in all experiments, since expression of this gene was found constant among samples. The amounts of viral RNA were determined by quantitative RT-PCR as described before [21].

### Microarray hybridizations

The microarray experiments were performed as described previously [5]. Briefly, mRNA was amplified from 1 μg of total RNA by cDNA synthesis with oligo(dT) double-anchored primers, followed by *in vitro *transcription using a T7 RNA polymerase kit (Ambion). During transcription, 5-(3-aminoallyl)-UTP was incorporated into the single stranded cRNA. Cy3 and Cy5 NHS-esters (Amersham Biosciences) were coupled to 2 μg cRNA. RNA quality was monitored after each successive step using the equipment described above. Corning UltraGAPS slides, printed with a Mouse Array-Ready Oligo set (Operon; 35,000 spots), were hybridized with 1 μg of each alternatively labeled cRNA target at 42°C for 16–20 h. Two independent dye-swap hybridizations (4 arrays) were performed for each experimental group. After hybridization the slides were washed extensively and scanned using the Agilent G2565AA DNA Microarray Scanner.

### Statistical analysis

After data extraction using Imagene 5.6 Software (BioDiscovery), Lowess normalization [57] was performed on mean spot-intensities in order to correct for dye and print-tip biases [58]. The microarray data was analysed using ANOVA (R version 2.2.1/MAANOVA version 0.98–7) [59]. Briefly, in a fixed effect analysis, sample, array and dye effects were modelled. P-values were determined by a permutation F2-test, in which residuals were shuffled 5,000 times globally. Genes with P < 0.05 after family wise error correction were considered significantly changed. Cluster analysis (standard correlation) was performed with GeneSpring GX 7.2 software (Silicon Genetics). When indicated, the confidence level was increased by applying a fold change cut-off. The resulting genelists were subjected to Genespring 7.2 software for further analysis.

### ArrayExpress accession numbers

MIAME-compliant data in MAGE-ML format as well as complete descriptions of protocols have been submitted to the public microarray database ArrayExpress  with the following accession numbers: microarray layout, P-UMCU-8; gene expression data of MHV-infected mice, E-MEXP-2081; protocols for total RNA isolation and mRNA amplification, P-MEXP-34397; cRNA labeling, P-MEXP-34400 and P-MEXP-35534; hybridization and washing of slides, P-MEXP-34401; scanning of slides, P-MEXP-34430; data normalization, P-MEXP-34431.

## Competing interests

The authors declare that they have no competing interests.

## Authors' contributions

MR and MJAGK conducted all the experiments. MR wrote the manuscript. PJMR and CAMdeH coordinated the research efforts and assisted with writing the manuscript. All authors read and approved the final manuscript.

## Supplementary Material

Additional file 1 **Gene expression profiles in the brain of MHV-infected mice**. (A) Early genes (n = 57), and (B) Late genes (n = 64). The induction of expression for each gene in infected animals relative to the PBS-inoculated animals is indicated for the different conditions (i.e. mouse strain and day post infection). Click here for file

Additional file 2 **Differentially expressed genes per BALB/c mouse**. The induction of gene expression at day 5 for 96 genes relative to the PBS-inoculated animals is indicated for the four individual BALB/c mice. Differential gene expression correlates with the viral load. Click here for file

Additional file 3 **Type I IFN-independent genes**. The induction of differential gene expression (≥ 1.5 fold) in the brain of MHV-infected IFNAR-/- mice at day 5 relative to the PBS-inoculated animals is indicated. The relative expression of these genes in the parental 129SvEv mice after infection with MHV is also shown. Click here for file

Additional file 4 **Tentative type I IFN-dependent genes**. List of genes the expression of which was upregulated (≥ 1.5 fold) in the BALB/c mice but not significantly changed in the IFNAR-/- mice upon infection with MHV. Genes, the transcriptional upregulation of which was at least 2 times higher in the BALB/c mice than in the IFNAR-/- mice, were also added to the list. Click here for file

Additional file 5 **Tentative type I IFN-dependent gene expression network**. The genes listed in Additional file 4 (n = 82) were subjected to functional association network analysis by using the public STRING 8.0 database . Indicated is the confidence view of the analysis. Stronger associations are symbolized by thicker lines. The central players in the network (i.e. Irf1 and Irf7) are indicated in red. Click here for file
