# Supplementary material for: Type I interferon receptor-independent and -dependent host transcriptional responses to mouse hepatitis coronavirus infection in vivo
Source: BMC Genomics. 2009 Aug 3;10:350. doi: 10.1186/1471-2164-10-350 (PMC2728740; doi:10.1186/1471-2164-10-350)
Supplement: Additional file 1 — Gene expression profiles in the brain of MHV-infected mice. (A) Early genes (n = 57), and (B) Late genes (n = 64). The induction of expression for each gene in infected animals relative to the PBS-inoculated animals is indicated for the different conditions (i.e. mouse strain and day post infection). [file 1471-2164-10-350-S1.pdf]

Supplementary Table 1A. Early genes (n=57)

GeneID	Common Name	RefSeq	BALB/c t=2	BALB/c t=5	129SvEv t=2	129SvEv t=5
MMAA300004126	69717	XM_001003852	**1,6**	**2,8**	1,0	1,0
MMAA300002620	1810023F06Rik	NM_029803	**4,4**	**10,4**	1,1	1,0
MMAA300008745	B2m	NM_009735	**2,2**	**5,4**	1,3	1,2
MMAA300017604	Bst2	NM_198095	**1,6**	**2,1**	1,0	1,0
MMAA200001340	C1qc	NM_007574	**1,5**	**1,9**	1,1	1,0
MMAA300009870	Ccl12	NM_011331	**1,7**	**9,3**	1,2	1,1
MMAA200009050	Cxcl11	NM_019494	**1,5**	**6,1**	1,0	1,0
MMAA300012414	D11Ertd759e	NM_001040005	**1,6**	**2,9**	1,4	1,0
MMAA200000807	D12Ertd647e	NM_026790	**2,7**	**3,0**	1,1	1,2
MMAA300004057	ENSMUSESTG00003733703		**1,8**	**2,6**	1,0	1,0
MMAA300012450	ENSMUSG00000039955		**1,9**	**3,2**	1,3	1,0
MMAA300012693	Fbxo39	NM_001037713	**2,0**	**2,8**	1,1	1,0
MMAA200000089	Gbp1	NM_010259	**2,7**	**6,1**	1,1	1,0
MMAA200004765	Gbp2	NM_010260	**2,4**	**4,1**	1,1	1,0
MMAA200000729	Gbp3	NM_018734	**3,5**	**5,5**	1,3	1,1
MMAA300006597	Gbp4	NM_029509	**5,3**	**4,8**	1,1	1,1
MMAA300017406	genomic:17-45870359-45870428		**6,1**	**8,3**	1,1	1,0
MMAA300011983	H28	NM_031367	**1,9**	**2,1**	1,1	1,0
MMAA300004062	H2-K1	NM_019909	**7,2**	**4,9**	1,1	1,2
MMAA300012864	H2-Q1		**1,7**	**3,2**	1,1	1,1
MMAA300010152	H2-Q10	NM_010391	**2,3**	**3,3**	0,9	1,0
MMAA300010147	H2-Q8	NM_023124	**1,6**	**2,2**	1,0	1,0
MMAA300006884	Herc5	XM_978982	**1,5**	**2,4**	1,0	1,0
MMAA300005162	Ifi202b	NM_008327	**2,2**	**7,2**	1,5	1,2
MMAA300005166	Ifi204	NM_008329	**1,5**	**5,2**	1,2	1,0
MMAA200006998	Ifi44	NM_133871	**4,3**	**6,4**	1,2	1,1
MMAA200004157	Ifih1	NM_027835	**1,6**	**2,8**	1,1	1,1
MMAA200002418	Ifit1	NM_008331	**3,7**	**3,4**	1,2	1,0
MMAA300016271	Ifit3	NM_010501	**5,9**	**7,6**	1,6	1,0
MMAA200009330	Ifitm3	NM_025378	**2,3**	**5,4**	1,8	1,1
MMAA200000303	Iigp2	NM_018738	**1,6**	**5,1**	1,2	1,0
MMAA200001244	Irf7	NM_016850	**1,6**	**2,2**	1,1	1,0
MMAA200006739	Irgm	NM_008326	**3,0**	**7,2**	1,1	1,0
MMAA200003721	Isg20	NM_020583	**2,4**	**3,3**	1,0	1,0
MMAA200000773	Isgf3g	NM_008394	**2,3**	**2,1**	1,0	1,0
MMAA200001213	Lgals3bp	NM_011150	**3,3**	**5,9**	1,4	1,1
MMAA200003577	Lgals9	NM_010708	**1,5**	**1,8**	1,0	1,0
MMAA200002687	Ly6c	NM_010738	**4,2**	**8,2**	1,8	1,0
MMAA200000279	Ly6e	NM_008529	**1,7**	**2,0**	0,9	1,1
MMAA200014954	Ly6f	NM_008530	**3,1**	**6,8**	1,2	1,0
MMAA200003282	Mx2	NM_013606	**2,0**	**2,6**	1,2	0,9
MMAA300000160	Oas1g	NM_011852	**2,4**	**6,2**	1,2	1,0
MMAA300006738	Oasl2	NM_011854	**2,8**	**4,5**	1,0	1,0
MMAA200004993	Olfr56	NM_010999	**2,5**	**6,9**	1,2	1,0
MMAA300011591	Parp12	NM_172893	**1,6**	**2,2**	1,1	1,0

MMAA200003254	Psmb8	NM_010724	**1,9**	**4,4**	1,1	1,1
MMAA200000293	Psme1	NM_011189	**4,0**	**2,1**	1,0	1,0
MMAA300018571	Samd9l	XM_983894	**1,8**	**4,1**	1,5	1,0
MMAA200002695	Stat1	NM_009283	**3,4**	**5,2**	1,1	1,0
MMAA300010868	Tap1	NM_013683	**1,7**	**5,3**	1,1	1,0
MMAA200003398	Tgtp	NM_001045540	**7,3**	**17,5**	1,9	1,1
MMAA200005563	Tor3a	NM_023141	**1,5**	**2,0**	1,0	1,0
MMAA200002067	Trim25	NM_009546	**1,9**	**2,6**	1,2	1,0
MMAA200000426	Ube1l	NM_023738	**2,1**	**2,9**	1,0	1,0
MMAA300005461	Ube2l6	NM_019949	**1,5**	**2,3**	1,0	0,9
MMAA200005576	Usp18	NM_011909	**1,9**	**3,9**	1,0	1,0
MMAA300005673	Zbp1	NM_021394	**2,7**	**6,2**	1,1	1,0

Supplementary Table 1B. Late genes (n=64)

GeneID	Common Name	RefSeq	BALB/c t=2	BALB/c t=5	129SvEv t=2	129SvEv t=5
MMAA300004129	15040	XM_992574	1,4	**1,9**	1,0	1,0
MMAA300007721	665536		1,1	**1,8**	1,1	1,0
MMAA300002618	1810023F06Rik	NM_029803	1,4	**2,1**	1,1	0,9
MMAA200003525	6620401K05Rik	NM_172774	1,1	**1,8**	1,1	1,0
MMAA300012210	AI481105	XM_989905	1,4	**1,7**	0,9	1,0
MMAA300006778	Arpc1b	NM_023142	0,9	**1,9**	1,0	1,1
MMAA200000131	C1qa	NM_007572	1,3	**2,3**	1,0	1,0
MMAA200000049	Ccl2	NM_011333	1,0	**7,5**	1,0	1,0
MMAA300009668	Ccl5	NM_013653	0,9	**2,8**	1,0	1,0
MMAA200003414	Ccl7	NM_013654	0,9	**2,8**	1,0	1,1
MMAA300004234	Cd74	NM_001042605	1,0	**2,0**	1,0	0,9
MMAA300000452	Cp	NM_007752	1,3	**2,8**	1,1	1,0
MMAA200000242	Ctsc	NM_009982	1,1	**2,5**	1,1	1,0
MMAA200001409	Ctss	NM_021281	1,2	**2,0**	1,1	1,0
MMAA200000311	Cxcl10	NM_021274	1,4	**13,0**	1,1	1,0
MMAA200000272	Cxcl9	NM_008599	1,0	**2,2**	1,0	1,0
MMAA300009934	D14Ertd668e	NM_199015	1,2	**2,4**	1,0	1,0
MMAA300018596	ENSMUSG00000063388		1,1	**2,7**	1,1	1,0
MMAA200004470	Fcer1g	NM_010185	1,0	**2,5**	1,1	1,1
MMAA200000053	Fcgr1	NM_010186	1,4	**2,1**	1,0	1,0
MMAA300021278	Fcgr3a	NM_144559	1,0	**2,1**	1,0	1,0
MMAA200003376	Gzma	NM_010370	0,9	**2,5**	1,0	1,0
MMAA200003391	H2-Ea	NM_010381	1,1	**2,1**	1,1	1,0
MMAA200014858	H2-Q1		1,1	**1,9**	1,0	1,1
MMAA300010153	H2-Q9	NM_010394	1,4	**2,4**	1,1	1,1
MMAA200000255	Hck	NM_010407	1,1	**1,9**	1,0	1,0
MMAA300010905	Icam1	NM_010493	1,0	**2,7**	1,0	1,0
MMAA300005165	Ifi205	NM_001033450	1,2	**2,1**	1,1	1,0
MMAA300004660	Ifitm1	NM_026820	1,0	**2,3**	1,0	1,0
MMAA200000196	Ifngr1	NM_010511	1,0	**1,6**	1,1	1,0
MMAA200000461	Irf1	NM_008390	1,4	**6,5**	1,1	1,0
MMAA200001229	Irf8	NM_008320	1,1	**1,7**	1,0	1,0
MMAA300015119	Krt18	NM_010664	1,2	**1,6**	0,9	1,1
MMAA300005306	Lcn2	NM_008491	1,4	**6,8**	1,0	1,1
MMAA300021034	Lgals3	NM_010705	1,3	**1,8**	1,0	1,0
MMAA300001891	Lilrb4	NM_013532	1,2	**1,8**	1,1	1,0
MMAA300002751	Ly86	NM_010745	0,8	**1,9**	1,0	1,0
MMAA300002048	Lyzs	NM_017372	0,9	**2,5**	1,0	1,0
MMAA300004266	Ms4a4b	NM_021718	0,9	**1,9**	1,0	1,0
MMAA200006504	Ms4a6b	NM_028595	1,1	**2,6**	0,9	1,0
MMAA200011666	Ms4a6d	NM_026835	1,1	**2,5**	1,1	1,0
MMAA200009417	Mt2	NM_008630	1,0	**1,7**	1,0	0,9
MMAA200007409	Myct1	NM_026793	0,9	**1,9**	1,1	1,0
MMAA200016125	Oasl1	NM_145209	1,4	**3,0**	1,1	1,0
MMAA200002994	Osmr	NM_011019	1,0	**1,7**	1,0	1,1

MMAA300009340	Parp14	NM_001039530	1,2	**2,2**	1,1	1,0
MMAA300015703	Phf11	NM_172603	1,4	**3,1**	1,2	1,0
MMAA200007618	Plac8	NM_139198	1,0	**2,9**	1,1	1,0
MMAA200013018	Plec1	XM_993494	1,3	**2,6**	1,0	1,0
MMAA200000278	Psmb10	NM_013640	1,2	**2,3**	1,0	1,0
MMAA200003424	Psmb9	NM_013585	1,4	**4,0**	1,1	1,0
MMAA300018890	Psme1	NM_011189	1,0	**2,1**	1,2	1,0
MMAA200003295	Saa3	NM_011315	1,0	**5,0**	1,0	1,0
MMAA200012660	Samhd1	NM_018851	1,3	**2,1**	1,0	1,0
MMAA200008133	Serping1	NM_009776	1,0	**2,2**	1,1	1,0
MMAA200000042	Socs1	NM_009896	1,1	**1,5**	1,2	1,0
MMAA200004256	Stat2	NM_019963	1,2	**1,9**	0,9	1,0
MMAA200003694	Tgm2	NM_009373	1,3	**2,5**	1,0	0,9
MMAA300000154	Timp1	NM_011593	1,0	**1,9**	1,0	1,0
MMAA200015882	Tlr2	NM_011905	1,0	**2,3**	1,1	1,0
MMAA200000616	Tspo	NM_009775	1,0	**2,0**	1,0	1,0
MMAA300002295	Tyki	NM_020557	1,4	**2,0**	1,0	1,0
MMAA300017681	XR_003396.1	XM_924014	1,3	**2,4**	1,1	1,1
MMAA300015120	XR_005070.1	XR_005070	1,1	**1,6**	1,0	1,0
