# Supplementary material for: Type I interferon receptor-independent and -dependent host transcriptional responses to mouse hepatitis coronavirus infection in vivo
Source: BMC Genomics. 2009 Aug 3;10:350. doi: 10.1186/1471-2164-10-350 (PMC2728740; doi:10.1186/1471-2164-10-350)
Supplement: Additional file 2 — Differentially expressed genes per BALB/c mouse. The induction of gene expression at day 5 for 96 genes relative to the PBS-inoculated animals is indicated for the four individual BALB/c mice. Differential gene expression correlates with the viral load. [file 1471-2164-10-350-S2.pdf]

Supplementary Table 2. Differentially expressed genes per BALB/c mouse (n=96)

GeneID	Common Name	RefSeq	Mouse 1	Mouse 2	Mouse 3	Mouse 4
MMAA300004126	69717	XM_001003852	3,3	1,7	4,6	1,5
MMAA300002620	1810023F06Rik	NM_029803	3,3	20,8	3,4	14,1
MMAA300008745	B2m	NM_009735	2,5	10,0	2,7	6,4
MMAA300017604	Bst2	NM_198095	2,2	2,4	2,0	2,0
MMAA200000131	C1qa	NM_007572	2,3	2,7	1,8	2,3
MMAA300009870	Ccl12	NM_011331	2,9	15,1	2,4	16,8
MMAA200000049	Ccl2	NM_011333	3,7	14,5	2,1	9,7
MMAA300009668	Ccl5	NM_013653	1,5	5,0	1,4	3,3
MMAA200003414	Ccl7	NM_013654	1,4	5,2	0,9	3,8
MMAA300004234	Cd74	NM_001042605	1,1	3,0	1,4	2,5
MMAA300000452	Cp	NM_007752	0,6	5,3	0,6	4,5
MMAA200000242	Ctsc	NM_009982	1,3	4,3	1,3	3,0
MMAA200001409	Ctss	NM_021281	1,9	2,5	1,7	1,9
MMAA200000311	Cxcl10	NM_021274	6,6	22,5	6,0	17,1
MMAA200009050	Cxcl11	NM_019494	3,2	9,8	2,9	8,3
MMAA200000272	Cxcl9	NM_008599	1,2	3,6	1,0	2,7
MMAA300012414	D11Ertd759e	NM_001040005	1,4	4,6	1,4	4,1
MMAA200000807	D12Ertd647e	NM_026790	3,6	2,3	4,1	2,2
MMAA300009934	D14Ertd668e	NM_199015	1,2	4,3	1,1	3,2
MMAA300004057	ENSMUSESTG00003733703		1,7	4,1	1,9	2,6
MMAA300012450	ENSMUSG00000039955		2,7	3,9	3,1	3,2
MMAA300018596	ENSMUSG00000063388		1,6	4,2	1,7	3,3
MMAA300012693	Fbxo39	NM_001037713	1,6	4,7	1,4	3,7
MMAA200004470	Fcer1g	NM_010185	2,1	3,1	2,0	2,6
MMAA200000053	Fcgr1	NM_010186	1,9	2,5	1,7	2,2
MMAA300021278	Fcgr3a	NM_144559	1,3	3,0	1,3	2,8
MMAA200000089	Gbp1	NM_010259	3,4	10,5	4,1	6,2
MMAA200004765	Gbp2	NM_010260	2,9	4,2	3,2	6,2
MMAA200000729	Gbp3	NM_018734	3,0	8,9	2,8	7,3
MMAA300006597	Gbp4	NM_029509	1,0	10,6	1,5	6,1
MMAA300017406	genomic:17-45870359-45870428		7,0	10,1	9,4	6,6
MMAA200003376	Gzma	NM_010370	1,0	5,2	1,1	2,8
MMAA300011983	H28	NM_031367	1,8	2,7	1,6	2,3
MMAA200003391	H2-Ea	NM_010381	0,9	3,8	1,1	2,6
MMAA300004062	H2-K1	NM_019909	3,1	7,8	3,5	5,3
MMAA300012864	H2-Q1		2,7	3,7	2,9	3,3
MMAA300010152	H2-Q10	NM_010391	3,7	2,4	5,8	1,3
MMAA300010147	H2-Q8	NM_023124	2,8	2,4	2,0	1,6
MMAA300010153	H2-Q9	NM_010394	2,0	3,0	2,4	2,2
MMAA300006884	Herc5	XM_978982	1,3	4,5	1,1	2,8
MMAA300010905	Icam1	NM_010493	2,9	2,3	3,1	2,4
MMAA300005162	Ifi202b	NM_008327	0,8	15,3	0,7	11,9
MMAA300005166	Ifi204	NM_008329	1,4	9,2	1,3	8,7
MMAA300005165	Ifi205	NM_001033450	1,5	3,7	0,8	2,2
MMAA200006998	Ifi44	NM_133871	2,1	11,9	1,8	10,1

MMAA200004157	Ifih1	NM_027835	1,2	4,6	1,3	4,0
MMAA200002418	Ifit1	NM_008331	2,4	4,6	2,3	4,5
MMAA300016271	Ifit3	NM_010501	2,9	13,3	2,7	11,3
MMAA300004660	Ifitm1	NM_026820	2,1	3,1	2,0	1,9
MMAA200009330	Ifitm3	NM_025378	3,4	7,9	4,0	6,3
MMAA200000303	Iigp2	NM_018738	3,2	7,4	3,1	6,5
MMAA200000461	Irf1	NM_008390	2,8	12,5	2,5	8,0
MMAA200001244	Irf7	NM_016850	2,0	2,4	2,5	2,0
MMAA200006739	Irgm	NM_008326	2,9	13,7	2,8	9,6
MMAA200003721	Isg20	NM_020583	5,3	1,9	3,8	2,2
MMAA200000773	Isgf3g	NM_008394	1,8	2,8	1,9	2,0
MMAA300005306	Lcn2	NM_008491	1,8	13,2	2,8	9,4
MMAA200001213	Lgals3bp	NM_011150	3,4	8,2	3,9	8,0
MMAA200002687	Ly6c	NM_010738	5,0	13,2	5,2	9,3
MMAA200000279	Ly6e	NM_008529	2,2	2,1	1,7	2,1
MMAA200014954	Ly6f	NM_008530	3,4	11,7	3,7	8,3
MMAA300002048	Lyzs	NM_017372	2,0	3,0	2,2	2,7
MMAA200006504	Ms4a6b	NM_028595	1,1	5,0	1,0	3,5
MMAA200011666	Ms4a6d	NM_026835	1,7	4,1	1,5	2,7
MMAA200003282	Mx2	NM_013606	1,3	4,4	1,1	3,5
MMAA300000160	Oas1g	NM_011852	3,2	9,9	3,9	7,7
MMAA200016125	Oasl1	NM_145209	1,7	4,9	1,4	4,0
MMAA300006738	Oasl2	NM_011854	5,8	2,7	7,2	2,3
MMAA200004993	Olfr56	NM_010999	3,2	13,3	1,9	9,3
MMAA300011591	Parp12	NM_172893	1,3	3,4	1,2	2,9
MMAA300009340	Parp14	NM_001039530	1,4	3,1	1,4	2,7
MMAA300015703	Phf11	NM_172603	1,0	5,8	0,7	4,8
MMAA200007618	Plac8	NM_139198	2,2	3,6	2,9	3,0
MMAA200013018	Plec1	XM_993494	1,4	4,0	1,6	3,4
MMAA200000278	Psmb10	NM_013640	1,8	3,4	1,7	2,2
MMAA200003254	Psmb8	NM_010724	3,0	7,2	3,1	4,3
MMAA200003424	Psmb9	NM_013585	1,6	7,4	1,7	5,2
MMAA200000293	Psme1	NM_011189	1,7	2,7	1,8	2,1
MMAA200003295	Saa3	NM_011315	1,7	9,8	2,2	6,2
MMAA300018571	Samd9l	XM_983894	1,5	8,8	1,1	5,1
MMAA200012660	Samhd1	NM_018851	0,7	3,9	0,6	3,2
MMAA200008133	Serping1	NM_009776	1,0	4,2	1,0	2,8
MMAA200002695	Stat1	NM_009283	2,4	8,4	2,7	7,5
MMAA300010868	Tap1	NM_013683	2,0	10,4	1,7	7,2
MMAA200003694	Tgm2	NM_009373	1,3	4,2	1,2	3,3
MMAA200003398	Tgtp	NM_001045540	10,1	26,7	10,7	22,5
MMAA200015882	Tlr2	NM_011905	1,3	3,4	1,3	3,1
MMAA200005563	Tor3a	NM_023141	1,4	2,4	2,0	2,2
MMAA200002067	Trim25	NM_009546	1,7	3,8	1,7	3,4
MMAA200000616	Tspo	NM_009775	2,0	2,6	1,9	1,6
MMAA300002295	Tyki	NM_020557	2,0	2,0	2,1	1,9
MMAA200000426	Ube1l	NM_023738	1,5	4,5	1,4	4,1
MMAA300005461	Ube2l6	NM_019949	1,6	3,2	1,5	2,8

MMAA200005576	Usp18	NM_011909	2,8	5,9	2,2	4,7
MMAA300017681	XR_003396.1	XM_924014	1,7	3,7	1,8	2,6
MMAA300005673	Zbp1	NM_021394	2,2	12,2	1,7	8,6
