# Supplementary material for: Type I interferon receptor-independent and -dependent host transcriptional responses to mouse hepatitis coronavirus infection in vivo
Source: BMC Genomics. 2009 Aug 3;10:350. doi: 10.1186/1471-2164-10-350 (PMC2728740; doi:10.1186/1471-2164-10-350)
Supplement: Additional file 3 — Type I IFN-independent genes. The induction of differential gene expression (≥ 1.5 fold) in the brain of MHV-infected IFNAR-/- mice at day 5 relative to the PBS-inoculated animals is indicated. The relative expression of these genes in the parental 129SvEv mice after infection with MHV is also shown. [file 1471-2164-10-350-S3.pdf]

Supplementary Table 3. Type I IFN-independent genes (n=73)

GeneID	Common Name	RefSeq	129SvEv t=2	129SvEv t=5	IFNAR t=2	IFNAR t=5
MMAA300018417	665509		1,1	1,0	1,1	**1,5**
MMAA200000796	Apod	NM_007470	1,2	1,0	1,0	**2,0**
MMAA300006778	Arpc1b	NM_023142	1,0	1,1	1,0	**1,6**
MMAA200004830	Arrdc2	NM_027560	1,0	1,0	1,0	**1,8**
MMAA300008745	B2m	NM_009735	1,3	1,2	1,0	**4,1**
MMAA200000131	C1qa	NM_007572	1,0	1,0	0,9	**2,1**
MMAA200000979	C1qb	NM_009777	1,0	1,0	1,0	**3,1**
MMAA200001340	C1qc	NM_007574	1,1	1,0	1,0	**2,0**
MMAA300004234	Cd74	NM_001042605	1,0	0,9	1,0	**3,8**
MMAA200007578	Cdkn1a	NM_007669	1,0	1,0	1,0	**2,1**
MMAA300012869	Chi3l3	XM_889251	1,1	1,0	1,0	**3,3**
MMAA300000452	Cp	NM_007752	1,1	1,0	0,9	**2,3**
MMAA200009050	Cxcl11	NM_019494	1,0	1,0	1,0	**1,6**
MMAA300001385	Cyp2j9	NM_028979	0,9	0,9	1,0	**1,6**
MMAA300012450	ENSMUSG00000039955		1,3	1,0	1,0	**2,1**
MMAA200001345	Flt1	NM_010228	1,1	1,1	1,0	**1,6**
MMAA200000089	Gbp1	NM_010259	1,1	1,0	1,0	**3,2**
MMAA200004765	Gbp2	NM_010260	1,1	1,0	1,0	**2,3**
MMAA200000729	Gbp3	NM_018734	1,3	1,1	1,1	**1,9**
MMAA200005744	Gdpd3	NM_024228	1,1	1,0	1,0	**1,6**
MMAA200000881	Glul	NM_008131	1,0	1,0	0,9	**1,6**
MMAA300020817	Grrp1	XM_485455	0,9	1,0	1,0	**1,5**
MMAA200000761	Gstm1	NM_010358	1,0	0,9	1,0	**1,5**
MMAA300010457	H2-Aa	NM_010378	1,3	0,9	1,0	**1,9**
MMAA300004076	H2-Eb1	NM_010382	1,1	1,0	1,0	**1,7**
MMAA300004062	H2-K1	NM_019909	1,1	1,2	1,0	**2,8**
MMAA300012864	H2-Q1		1,1	1,1	1,0	**1,7**
MMAA300010153	H2-Q9	NM_010394	1,1	1,1	1,0	**1,6**
MMAA200005712	Hexb	NM_010422	1,1	1,0	1,1	**1,5**
MMAA300017926	Hspb1	NM_013560	1,1	1,0	1,1	**1,6**
MMAA300005162	Ifi202b	NM_008327	1,5	1,2	1,3	**1,5**
MMAA300004660	Ifitm1	NM_026820	1,0	1,0	1,1	**2,2**
MMAA200009330	Ifitm3	NM_025378	1,8	1,1	1,1	**3,0**
MMAA200000196	Ifngr1	NM_010511	1,0	1,0	1,0	**1,6**
MMAA200000191	Igfbp7	NM_008048	1,0	1,0	1,1	**2,1**
MMAA200000303	Iigp2	NM_018738	1,2	1,0	1,0	**1,8**
MMAA200000461	Irf1	NM_008390	1,1	1,0	1,1	**1,9**
MMAA300015119	Krt18	NM_010664	0,9	1,1	1,0	**2,2**
MMAA300005306	Lcn2	NM_008491	1,0	1,1	1,1	**2,9**
MMAA200001213	Lgals3bp	NM_011150	1,4	1,1	1,0	**1,5**
MMAA200011815	Lrg1	NM_029796	1,0	1,0	1,1	**1,6**
MMAA200002687	Ly6c	NM_010738	1,8	1,0	1,0	**6,6**
MMAA200014954	Ly6f	NM_008530	1,2	1,0	1,0	**3,4**
MMAA300002048	Lyzs	NM_017372	1,0	1,0	1,0	**4,2**
MMAA300002047	Lzp-s	NM_013590	1,0	1,0	1,0	**1,8**

MMAA300007075	Mgp	NM_008597	1,1	1,0	1,0	**1,9**
MMAA200006504	Ms4a6b	NM_028595	0,9	1,0	1,0	**1,6**
MMAA300007885	Mt1	NM_013602	1,0	0,9	1,1	**2,5**
MMAA200009417	Mt2	NM_008630	1,0	0,9	1,0	**2,3**
MMAA300007883	Mt3	NM_013603	1,1	0,9	1,2	**1,5**
MMAA200004993	Olfr56	NM_010999	1,2	1,0	1,0	**2,2**
MMAA300000072	Phyhd1	NM_177725	1,0	1,0	1,2	**3,1**
MMAA200007618	Plac8	NM_139198	1,1	1,0	1,0	**1,5**
MMAA200016029	Podxl	NM_013723	1,1	1,0	1,1	**1,6**
MMAA200003254	Psmb8	NM_010724	1,1	1,1	1,0	**2,1**
MMAA200003424	Psmb9	NM_013585	1,1	1,0	1,0	**2,0**
MMAA300005843	S100a11	NM_016740	1,0	1,1	1,2	**1,6**
MMAA200003295	Saa3	NM_011315	1,0	1,0	1,1	**2,0**
MMAA200003167	Scrg1	NM_009136	1,0	1,0	1,0	**1,5**
MMAA200003360	Serpina3g	NM_009251	1,0	1,0	1,0	**1,8**
MMAA300001921	Sgk	NM_011361	0,9	1,1	1,0	**1,5**
MMAA300006210	Slc2a1	NM_011400	1,0	1,0	1,0	**1,6**
MMAA300004422	Sorbs1	NM_178362	1,1	1,0	1,0	**1,5**
MMAA200002695	Stat1	NM_009283	1,1	1,0	1,0	**1,5**
MMAA200003495	Sult1a1	NM_133670	0,9	1,1	1,1	**3,3**
MMAA200003694	Tgm2	NM_009373	1,0	0,9	1,0	**2,5**
MMAA200003398	Tgtp	NM_001045540	1,9	1,1	1,0	**13,1**
MMAA200006098	Tm2d2	NM_027194	1,1	0,9	1,3	**2,7**
MMAA300007712	Tsc22d3	NM_010286	1,0	1,0	1,0	**2,0**
MMAA200009290	Ubd	NM_023137	1,0	1,0	1,0	**1,8**
MMAA300017681	XR_003396.1	XM_924014	1,1	1,1	1,0	**1,9**
MMAA300015120	XR_005070.1	XR_005070	1,0	1,0	1,0	**1,8**
MMAA200003752	Zfp113	NM_019747	1,0	1,0	1,0	**1,5**
