# Supplementary material for: Type I interferon receptor-independent and -dependent host transcriptional responses to mouse hepatitis coronavirus infection in vivo
Source: BMC Genomics. 2009 Aug 3;10:350. doi: 10.1186/1471-2164-10-350 (PMC2728740; doi:10.1186/1471-2164-10-350)
Supplement: Additional file 4 — Tentative type I IFN-dependent genes. List of genes the expression of which was upregulated (≥ 1.5 fold) in the BALB/c mice but not significantly changed in the IFNAR-/- mice upon infection with MHV. Genes, the transcriptional upregulation of which was at least 2 times higher in the BALB/c mice than in the IFNAR-/- mice, were also added to the list. [file 1471-2164-10-350-S4.pdf]

Supplementary Table 4. Tentative type I IFN-dependent genes (n=82)

GeneID	Common Name	RefSeq	BALB/c t=5	IFNAR t=5	Ratio
MMAA300004129	15040	XM_992574	**1,9**	1,0	1,8
MMAA300004126	69717	XM_001003852	**2,5**	1,0	2,5
MMAA300002618	1810023F06Rik	NM_029803	**1,8**	1,1	1,6
MMAA200003525	6620401K05Rik	NM_172774	**1,7**	1,1	1,6
MMAA300012210	AI481105	XM_989905	**1,7**	0,9	1,8
MMAA300017604	Bst2	NM_198095	**2,1**	1,0	2,1
MMAA300009870	Ccl12	NM_011331	**6,5**	1,4	4,6
MMAA200000049	Ccl2	NM_011333	**5,8**	1,1	5,1
MMAA300009668	Ccl5	NM_013653	**2,4**	1,1	2,1
MMAA200003414	Ccl7	NM_013654	**2,3**	1,0	2,3
MMAA200000242	Ctsc	NM_009982	**2,2**	1,3	1,7
MMAA200000311	Cxcl10	NM_021274	**11,1**	1,3	8,6
MMAA200009050	Cxcl11	NM_019494	**6,1**	**1,6**	3,7
MMAA200000272	Cxcl9	NM_008599	**1,9**	1,1	1,7
MMAA300012414	D11Ertd759e	NM_001040005	**2,4**	1,2	2,1
MMAA200000807	D12Ertd647e	NM_026790	**2,9**	1,0	2,8
MMAA300009934	D14Ertd668e	NM_199015	**2,0**	1,0	2,0
MMAA300004057	ENSMUSESTG00003733703		**2,4**	1,4	1,8
MMAA300012693	Fbxo39	NM_001037713	**2,5**	1,0	2,4
MMAA200000053	Fcgr1	NM_010186	**2,1**	1,1	1,9
MMAA300021278	Fcgr3a	NM_144559	**2,0**	1,1	1,8
MMAA200000729	Gbp3	NM_018734	**5,5**	**1,9**	2,9
MMAA300006597	Gbp4	NM_029509	**3,1**	1,2	2,7
MMAA300017406	genomic:17-45870359-45870428		**8,1**	1,1	7,3
MMAA200003376	Gzma	NM_010370	**2,0**	1,0	2,0
MMAA300011983	H28	NM_031367	**2,0**	1,0	2,0
MMAA200003391	H2-Ea	NM_010381	**1,8**	1,1	1,6
MMAA300004061	H2-K1	NM_019909	**3,3**	1,2	2,7
MMAA300010152	H2-Q10	NM_010391	**2,9**	1,2	2,5
MMAA300010147	H2-Q8	NM_023124	**2,2**	1,0	2,2
MMAA300006884	Herc5	XM_978982	**2,1**	1,1	1,9
MMAA300010905	Icam1	NM_010493	**2,7**	1,4	1,8
MMAA300005162	Ifi202b	NM_008327	**7,2**	1,5	4,8
MMAA300005166	Ifi204	NM_008329	**3,5**	1,1	3,2
MMAA300005165	Ifi205	NM_001033450	**1,8**	1,1	1,7
MMAA200006998	Ifi44	NM_133871	**4,6**	1,1	4,2
MMAA200004157	Ifih1	NM_027835	**2,3**	1,0	2,2
MMAA200002418	Ifit1	NM_008331	**3,3**	1,2	2,7
MMAA300016271	Ifit3	NM_010501	**5,9**	1,2	4,8
MMAA200000303	Iigp2	NM_018738	**5,1**	**1,8**	2,8
MMAA200000461	Irf1	NM_008390	**6,5**	**1,9**	3,5
MMAA200001244	Irf7	NM_016850	**2,2**	1,1	2,1
MMAA200003721	Isg20	NM_020583	**3,0**	1,0	3,1
MMAA200000773	Isgf3g	NM_008394	**2,1**	1,1	1,9
MMAA300005306	Lcn2	NM_008491	**6,8**	**2,9**	2,3

MMAA300021034	Lgals3	NM_010705	**1,8**	1,1	1,7
MMAA200001213	Lgals3bp	NM_011150	**5,9**	1,5	3,8
MMAA200003577	Lgals9	NM_010708	**1,8**	1,1	1,6
MMAA300001891	Lilrb4	NM_013532	**1,7**	1,1	1,5
MMAA200000279	Ly6e	NM_008529	**2,0**	1,2	1,7
MMAA200014954	Ly6f	NM_008530	**6,8**	**3,4**	2,0
MMAA300004266	Ms4a4b	NM_021718	**1,9**	1,0	1,8
MMAA200006504	Ms4a6b	NM_028595	**2,6**	1,6	1,7
MMAA200011666	Ms4a6d	NM_026835	**2,3**	1,1	2,1
MMAA200003282	Mx2	NM_013606	**2,2**	1,0	2,2
MMAA200007409	Myct1	NM_026793	**1,7**	1,0	1,7
MMAA300000160	Oas1g	NM_011852	**5,5**	1,1	5,0
MMAA200016125	Oasl1	NM_145209	**2,6**	1,0	2,7
MMAA300006738	Oasl2	NM_011854	**4,0**	1,0	4,1
MMAA200004993	Olfr56	NM_010999	**6,9**	**2,2**	3,2
MMAA300011591	Parp12	NM_172893	**2,0**	1,1	1,8
MMAA300009340	Parp14	NM_001039530	**2,0**	1,1	1,8
MMAA300015703	Phf11	NM_172603	**2,1**	1,2	1,7
MMAA200007618	Plac8	NM_139198	**2,9**	1,5	2,0
MMAA200013018	Plec1	XM_993494	**2,3**	1,3	1,8
MMAA200000278	Psmb10	NM_013640	**2,2**	1,0	2,1
MMAA200003254	Psmb8	NM_010724	**4,4**	**2,1**	2,1
MMAA200003424	Psmb9	NM_013585	**4,0**	**2,0**	2,0
MMAA300003147	Psme1	NM_011189	**1,9**	1,0	1,8
MMAA200003295	Saa3	NM_011315	**5,0**	**2,0**	2,5
MMAA300018571	Samd9l	XM_983894	**3,0**	1,2	2,5
MMAA200002695	Stat1	NM_009283	**5,2**	**1,5**	3,6
MMAA200004256	Stat2	NM_019963	**1,9**	1,0	1,9
MMAA300000154	Timp1	NM_011593	**1,9**	1,0	1,8
MMAA200015882	Tlr2	NM_011905	**2,1**	1,1	1,9
MMAA200005563	Tor3a	NM_023141	**2,0**	1,1	1,9
MMAA200002067	Trim25	NM_009546	**2,5**	1,2	2,1
MMAA200000616	Tspo	NM_009775	**2,0**	1,2	1,6
MMAA300002295	Tyki	NM_020557	**2,0**	0,9	2,1
MMAA200000426	Ube1l	NM_023738	**2,5**	1,1	2,3
MMAA300005461	Ube2l6	NM_019949	**2,2**	1,1	2,1
MMAA200005576	Usp18	NM_011909	**3,6**	0,9	3,8
MMAA300005673	Zbp1	NM_021394	**4,4**	1,2	3,6
