# Supplementary figures and images for: Type I interferon receptor-independent and -dependent host transcriptional responses to mouse hepatitis coronavirus infection in vivo
Source: BMC Genomics. 2009 Aug 3;10:350. doi: 10.1186/1471-2164-10-350 (PMC2728740; doi:10.1186/1471-2164-10-350)

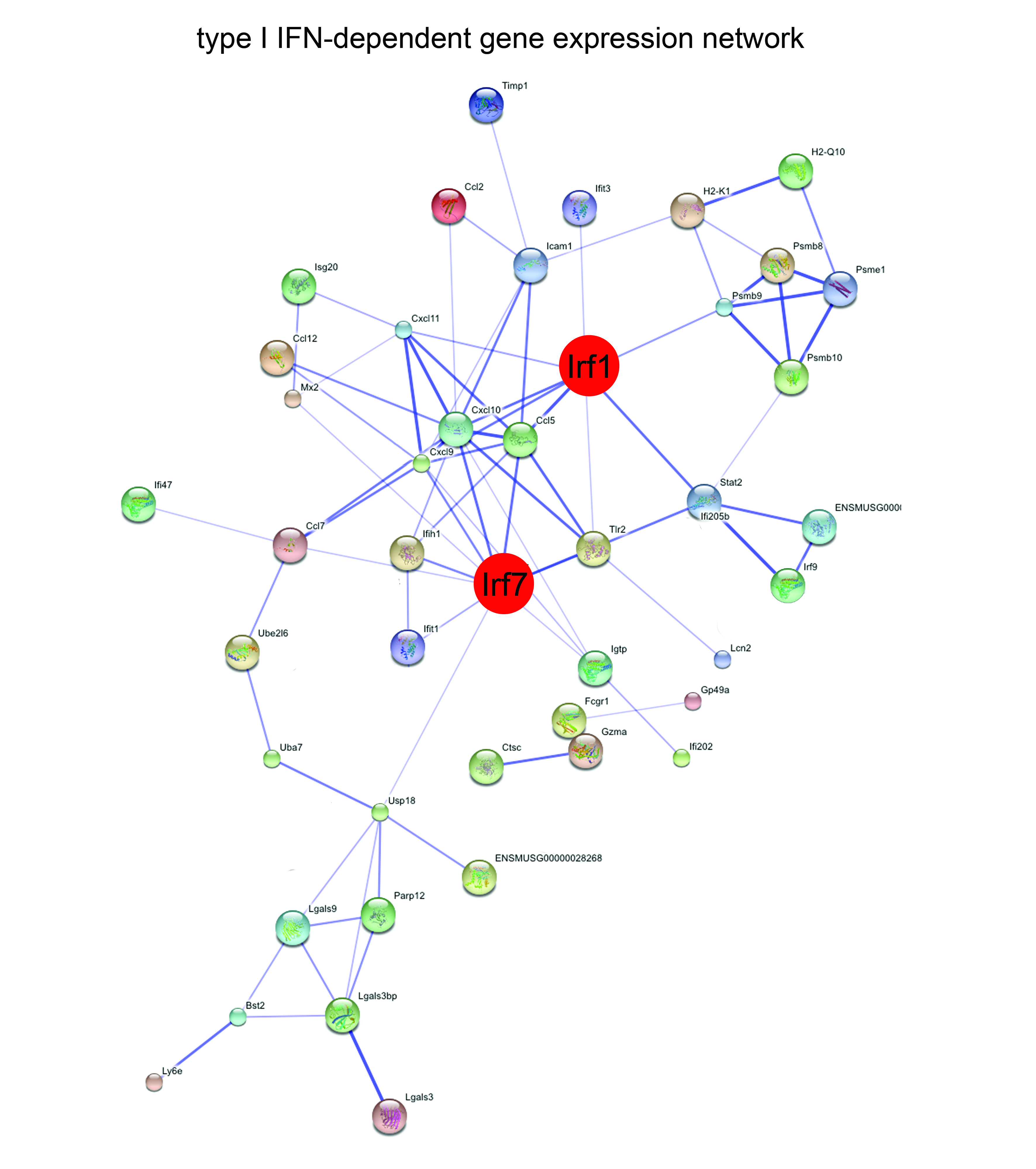

Supplement: Additional file 5 — Tentative type I IFN-dependent gene expression network. The genes listed in Additional file 4 (n = 82) were subjected to functional association network analysis by using the public STRING 8.0 database . Indicated is the confidence view of the analysis. Stronger associations are symbolized by thicker lines. The central players in the network (i.e. Irf1 and Irf7) are indicated in red. [file 1471-2164-10-350-S5.tiff]
